# Hydrophobicity of protein determinants influences the recognition of substrates by EDEM1 and EDEM2 in human cells

**DOI:** 10.1186/s12860-015-0047-7

**Published:** 2015-02-06

**Authors:** Iwona Sokołowska, Ewa S Piłka, Kirsten Sandvig, Grzegorz Węgrzyn, Monika Słomińska-Wojewódzka

**Affiliations:** Department of Molecular Biology, University of Gdańsk, Wita Stwosza 59, 80-308, Gdańsk, Poland; Evotec Ltd, Abingdon, UK; Department of Biochemistry, Institute for Cancer Research, The Norwegian Radium Hospital, Oslo University Hospital, Norway; and Centre for Cancer Biomedicine, University of Oslo, Oslo, Norway

**Keywords:** Endoplasmic reticulum, ERAD, EDEM proteins, Ricin, BACE457

## Abstract

**Background:**

EDEM1 and EDEM2 are crucial regulators of the endoplasmic reticulum (ER)-associated degradation (ERAD) that extracts misfolded glycoproteins from the calnexin chaperone system. The degradation of ERAD substrates involves mannose trimming of N-linked glycans; however the precise mechanism of substrate recognition and sorting to the ERAD pathway is still poorly understood. It has previously been demonstrated that EDEM1 and EDEM2 binding does not require the trimming of substrate glycans or even ERAD substrate glycosylation, thus suggesting that both chaperones probably recognize misfolded regions of aberrant proteins.

**Results:**

In this work, we focused on the substrate recognition by EDEM1 and EDEM2, asking whether hydrophobicity of protein determinants might be important for these interactions in human cells. In the study we used ricin, a protein toxin that utilizes the ERAD pathway in its retrotranslocation from the ER to the cytosol, and a model misfolded protein, the pancreatic isoform of human β-secretase, BACE457. Mutations in the hydrophobic regions of these proteins allowed us to obtain mutated forms with increased and decreased hydrophobicity.

**Conclusions:**

Our data provide the first evidence that recognition of ERAD substrates by EDEM1 and EDEM2 might be determined by a sufficiently high hydrophobicity of protein determinants. Moreover, EDEM proteins can bind hydrophobic transmembrane regions of misfolded ERAD substrates. These data contribute to the general understanding of the regulation of ERAD in mammalian cells.

**Electronic supplementary material:**

The online version of this article (doi:10.1186/s12860-015-0047-7) contains supplementary material, which is available to authorized users.

## Background

The endoplasmic reticulum (ER) plays a major role in the synthesis and folding of proteins. Among them, there are proteins operating at the plasma membrane or in the intracellular compartments, such as the ER, the endosomes, the lysosomes and the Golgi apparatus [1]. Acquisition of the protein’s native structure may fail or may progress unacceptably slowly, thus the ER is equipped with a quality control mechanism that discriminates correctly folded proteins from misfolded polypeptides [2]. As nascent proteins emerge from the ER translocation channel, the N-glycan Glc_3_Man_9_GlcNAc_2_ is attached to Asn residues. Following trimming by glucosidases I and II, monoglucosylated N-glycans can associate with the lectin-chaperones calnexin and calreticulin which assist protein folding [3,4]. The entry of proteins into the monoglucose cycle is regulated by removal and readdition of the final glucose by glucosidase II and UDP-glucose:glycoprotein glucosyl transferase, respectively. Properly folded proteins leave the ER along the secretory pathway [3,4]. Irreversibly misfolded proteins are sorted and degraded to neutralize their potential toxicity. This pathway is termed ER-associated degradation (ERAD) and is triggered by ER stress [5,6]. It results in retrotranslocation of misfolded proteins into the cytosol, followed by polyubiquitylation and proteasomal degradation. Three ER degradation-enhancing α-mannosidase I-like proteins (EDEM1, EDEM2, EDEM3) have been implicated in disposal of misfolded glycoproteins from the ER [7]. They belong to the glycoside hydrolase 47 family (GH47) comprising also the ER α1,2 mannosidase I and the Golgi α1,2 mannosidases. EDEMs are thought to function as lectins. It has been demonstrated that overexpression of recombinant EDEM1 [8-10] and EDEM2 [11,12] accelerates release of terminally misfolded glycoproteins from the calnexin chaperone system thereby enhancing ERAD by terminating glycoprotein’s folding attempts. Moreover, it is believed that in the mammalian ER, removal of several mannose residues is necessary to elicit disposal from the ER of misfolded glycoproteins. In this case Man_7-5_GlcNAc_2_ glycan forms are generated [13]. There is an ongoing debate whether the EDEM proteins are active mannosidases. Certainly, they possess the evolutionary conserved structure of the catalytic site as well as all the catalytic residues present in the other mannosidases of the GH47 family [14,15]. It has been demonstrated that EDEM1 can remove mannose residues from branch C [16] and as suggested also from branch A of glycoproteins [17]. EDEM3 can also enhance de-mannosylation of ERAD substrates [18]. Moreover, it has recently been demonstrated that EDEM2 initiates mammalian glycoprotein ERAD by catalyzing the first mannose trimming step and then mannose trimming from Man8GlcNAc2 to Man7GlcNAc2 is performed by EDEM3 and to a lesser extent also by EDEM1 [19]. Except glycan recognition, it has been shown that EDEM1 and EDEM2 can recognize protein substrates also in a glycan-independent manner [20-24]. Additionally, interactions of both lectins with proteins may depend on the structure of the substrate [23,24]. This was demonstrated during studies of the protein toxin ricin and the mechanisms of its retrotranslocation from the ER to the cytosol [23,24].

Ricin is a natural, extremely potent protein toxin isolated from castor beans, the seeds of the castor plant, *Ricinus communis*. Ricin holotoxin is a heterodimeric protein that consists of two polypeptide chains (A and B) joined by a disulfide bond [25]. The A-chain (RTA) inhibits protein synthesis by irreversibly inactivating eukaryotic ribosomes (for review see e.g., [26,27]). The B-chain (RTB) is a lectin, which binds to β-1,4-linked galactose residues. After binding to the cell surface, ricin is endocytosed and then transported retrogradely through the Golgi to the ER (for review see e.g. [28]). In the ER, the disulfide bond connecting the ricin A-chain and the B-chain is reduced [29], followed by a partial unfolding of RTA, rendering it competent to cross the ER membrane. Ricin A-chain is transported to the cytosol presumably via the Sec61p translocon in a similar manner as misfolded ER proteins [22,30]. Interaction between ricin and Sec61α protein, the main component of the Sec61p ER translocation channel was demonstrated by co-immunoprecipitation studies [22,30] and found in studies with isolated yeast ER-derived microsomes [31]. It was reported that retrograde translocation of ricin A-chain was dependent on a functional Sec61p complex [31]. Despite the fact that ricin is not a typical ERAD substrate it can utilize specific components of ER quality control to be translocated to the cytosol. In addition to interaction with EDEM1 [22,23] and EDEM2 [24], ricin A-chain requires SEL1L, a component of the mammalian HRD1 E3 ubiquitin ligase complex that takes part in the recognition of ER degradation substrates [32-34]. Interestingly, it has recently been demonstrated that in contrast to Shiga toxin, chaperone protein BiP (Grp78) negatively regulates ricin A-chain translocation from the ER to the cytosol [35]. Ricin A-chain contains a 12-amino-acid hydrophobic region (Val245 to Val256), which is located close to its C-terminus. This region is hidden in the holotoxin but becomes exposed upon A and B chain dissociation in the ER. It is possible that exposure of the RTA hydrophobic region in the ER triggers an interaction between ricin A-chain and membranes, ER chaperones or even ER translocons. It has been demonstrated that substitution of proline 250 into alanine (P250A) in this region results in a dramatic decrease in RTA_P250A_ cytotoxicity in Vero and HEK293 (human embryonic kidney) cells [23,36]. Decreased cytotoxicity of P250A ricin was explained by increased endosomal-lysosomal degradation of the toxin as well as reduced transport from the ER to the cytosol [23]. Moreover, transport of modified RTA to the cytosol, in contrast to wild-type RTA, appeared to be EDEM1- and EDEM2- independent [23,24]. The introduced P250A mutation alters the secondary structure of RTA into a more helical structure suggesting that EDEMs recognition might be determined by the structure of the ERAD substrate. Recognition of protein substrates by ER chaperones remains poorly defined. In the case of EDEM proteins it has been suggested that besides glycans, EDEM1 can recognize misfolded regions of aberrant proteins [19] such as exposed hydrophobic domains [10,37]. In this study, we have verified that changed hydrophobicity of protein determinants may influence protein substrate interaction with EDEM1 and EDEM2. We have demonstrated that ricin A-chain and a model misfolded protein, BACE457 carrying mutations that significantly decreased their hydrophobicity, are poorly recognized by EDEM1 and EDEM2 in contrast to their wild-type counterparts. Moreover, degradation of mutated BACE457 with decreased hydrophobicity is significantly reduced. Thus, we conclude that the chaperone proteins EDEM1 and EDEM2, similarly to the so called ‘classical chaperones’, recognize hydrophobic regions of the protein substrates and interact with determinants that posses relatively high degree of hydrophobicity.

## Results

### Biophysical characteristics of RTA mutants

**Figure 1:**
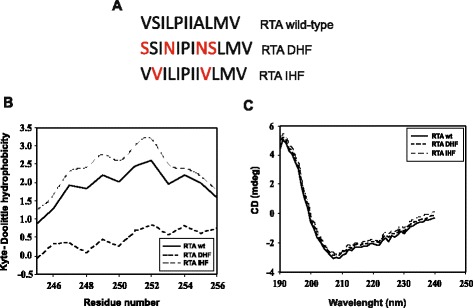
**Biochemical characteristics of RTA**_**DHF**_ **and RTA**_**IHF**_ **. (A)** Sequence of the C-terminal hydrophobic region of ricin A-chain. Introduced mutations are marked in red. **(B)** Hydrophobicity plot for the C-terminal region (Val245-Val256) of wild-type ricin A-chain, wt, DHF ricin A-chain and IHF ricin A-chain. Presented graphs are fragments of the output from ProtScale [64] prepared for full-length ricin A-chains. A sliding window of 13 AAs was used. Wild-type RTA (unbroken line); RTA_DHF_ (dashed line); RTA_IHF_ (dotted line) **(C)** Far UV CD spectra of wild-type RTA, wt; RTA_DHF_ and RTA_IHF_. The scans were corrected by the subtraction of blanks containing only the buffer. Averaged spectra from three measurements; s.d. ≤ 3%. Wild-type RTA (unbroken line); RTA_DHF_ (dashed line); RTA_IHF_ (dotted line).

To elucidate the significance of changed hydrophobicity of ricin A-chain on its intracellular transport and interaction with EDEM1 and EDEM2 proteins, we cloned appropriate genes with certain mutations and purified mutated forms of RTA containing amino acid substitutions in the C-terminal hydrophobic region of the ricin A-chain. It has been demonstrated before that this region is important in ricin cytotoxicity [23,36]. The purpose of the mutagenesis was to change the hydrophobicity of the desired region but to retain the secondary structure and mostly unaltered tertiary structure of the ricin A-chain. Detailed analysis based on the crystal structure of RTA [38] allowed us to select particular amino acids to be substituted (Table 1). This analysis showed that in the highly hydrophobic C-terminal region of ricin A-chain four residues can be substituted into hydrophilic residues without affecting RTA structure and its interaction with ricin B-chain (Table 1). Kyte*-*Doolittle hydropathy analysis revealed significant decrease in the hydrophobicity of this region (Figure 1A, B). On the other hand, two residues present in this region can be substituted into more hydrophobic residues increasing overall hydrophobicity of this region (Table 1, Figure 1A, B). Based on this information, we produced ricin A-chain with decreased hydrophobicity in the C-terminal region (referred as RTA_DHF_, see Methods) (Figure 1A, B) containing V245S, L248N, I252N, A253S substitutions. Similarly, the two substitutions (S246V, A253V) were introduced by site-directed mutagenesis to produce ricin A-chain with increased hydrophobicity in the C-terminal region (referred as RTA_IHF_, see Methods) (Figure 1A, B). Secondary structures of RTA_DHF_ and RTA_IHF_ were examined by circular dichroism (CD) and compared with the spectrum for unmodified RTA. Spectra for both RTA mutants indicate no significant alternations in their secondary structure (Figure 1C). The predicted models of RTA_DHF_ and RTA_IHF_ designed by Geno3D Server [39] did not show any significant changes in their tertiary structure when compared to the model of wild-type ricin A-chain (see Additional file 1A, B), what confirms our previous *in silico* analysis. Additionally, there are no alternations in the localization of the mutated hydrophobic regions of ricin A-chain within the protein (see Additional file 1A, B) and there are no significant changes in the surface charge distribution between wild-type RTA and mutated forms, DHF and IHF (see Additional file 1C).

**Table 1:** **Mutational analysis of the C-terminal hydrophobic region (Val245 to Val256) of ricin A-chain**

**RTA decreased hydrophobicity**				
**Position in RTA, C-terminal region**	**Primary residue**	**Altered residue**		**Comments**
		**Uncharged**	**Charged**	
245	Val (V)	Ser (S)	-	**Suggested**
247	Ile (I)	Asn (N)	Asp (D)	Not recommended, might disrupt RTA fold
248	Leu (L)	Asn (N)	Asp (D)	Asp may change RTA interactions, **Asn suggested**
249	Ile (I)	Asn (N)	Asp (D)	Not recommended, might disrupt fold and affect interaction with B-chain
250	Pro (P)	Ala (A)	-	Not recommended, Ala changes the structure of RTA [23]
251	Ile (I)	Asn (N)	Asp (D)	Not recommended, affects interaction with B-chain
252	Ile (I)	Asn (N)	Asp (D)	Asp may change RTA interactions, **Asn suggested**
253	Ala (A)	Ser (S)	-	**Suggested**
254	Leu (L)	Asn (N)	Asp (D)	Not recommended, might disrupt RTA fold
255	Met (M)	Gln (Q)	Lys (L)	Not recommended, might disrupt RTA fold
256	Val (V)	Ser (S)	-	Not recommended, might disrupt RTA fold
**RTA increased hydrophobicity**				
**Position in RTA, C-terminal region**	**Primary residue**	**Altered residue**		**Comments**
		**Uncharged**	**Charged**	
246	Ser (S)	Val (V)	-	**Suggested**
253	Ala (A)	Val (V)	-	**Suggested**

Mutations that were chosen to be introduced into this region are marked in bold.

**Figure 2:**
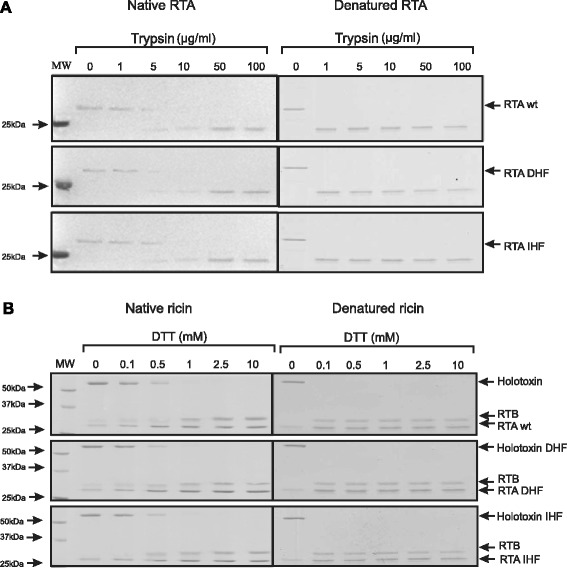
**Stability and reduction possibilities of DHF ricin and IHF ricin. (A)** Coomasie Blue-stained 12% SDS/PAGE gels showing the effect of the incubation of 500 ng of wild-type ricin A-chain, RTA wt, and modified ricin A-chains: RTA_DHF_ and RTA_IHF_, with increasing concentrations of trypsin. Digestion patterns for denatured forms of RTA are also shown. **(B)** Coomasie Blue-stained 12% SDS/PAGE gels showing the effect of increasing concentrations of dithriothreitol (DTT) on the reduction of native and denatured forms of wild-type holotoxin, DHF holotoxin and IHF holotoxin.

To characterize the correct folding and overall stability of RTA_DHF_ and RTA_IHF_, their sensitivity to trypsin (Figure 2A) and pronase (see Additional file 2) was compared to that of wild-type RTA. Digestion patterns for the ricin A-chain mutants and wild-type counterpart were the same, confirming equal *in vitro* stability of mutant RTA_DHF,_ RTA_IHF_ and the wild-type protein (Figure 2A, see Additional file 2). The digestion patterns significantly differ for denatured forms of ricin A-chain (Figure 2A, see Additional file 2) what further confirms high stability of native forms of RTA.

The obtained modified ricin A-chain proteins were reassociated with ricin B-chain to form a holotoxin (RTA_DHF_:RTB, RTA_IHF_:RTB; see Methods). To ensure that the introduced mutations were not affecting the reduction of the disulfide bond, either by increasing or decreasing the possibility of reductive cleavage, mutant RTA_DHF,_ RTA_IHF_ and wild-type holotoxin were incubated with increasing amounts of dithiothreitol (DTT). The results showed no significant difference in the RTA mutants reducibility in comparison to wild-type holotoxin (Figure 2B). The denatured forms of ricin were reduced by much lower concentrations of reducing agent (Figure 2B), what also indicate that native forms of ricin holotoxin, wild-type and mutant forms DHF and IHF are properly folded.

### The ricin A-chain mutants are not toxic to HEK293 cells

**Figure 3:**
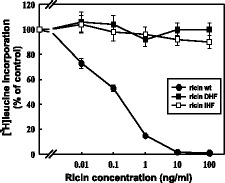
**DHF ricin and IHF ricin are not toxic to HEK293 cells.** Effect of increasing concentrations of DHF ricin, IHF ricin and wild-type ricin on HEK293 cells. Wild-type ricin, wt, (closed circles); modified ricin: DHF, (closed squares), IHF, (open squares). Data with s.d. from five independent experiments.

**Figure 4:**
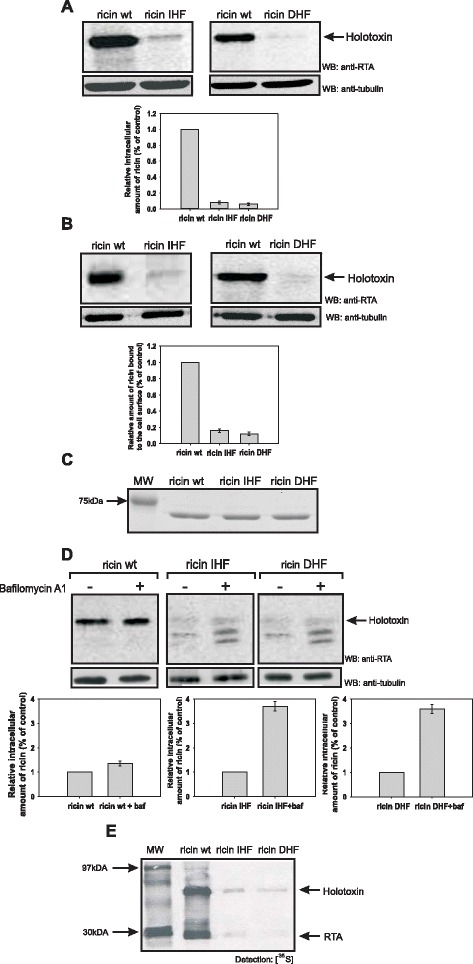
**DHF ricin and IHF ricin show decreased binding to the cell surface and increased degradation in low pH vesicles. (A)** HEK293 cells after 3 hours incubation with wild-type ricin, IHF ricin or DHF ricin run on SDS/PAGE under non-reducing conditions. A representative membranes after Western blot with anti-RTA antibodies are shown. Western blots with anti-tubulin antibodies were performed to show equal loading control. Data from three independent experiments are presented. RTA wt is marked as 1, other results are relative to this control. **(B)** Cells were incubated with wild-type ricin, IHF ricin or DHF ricin for 30 min at 0°C. Binding was measured as described in Methods. A representative membranes after Western blot with anti-RTA antibodies are shown. Western blots with anti-tubulin antibodies were performed to show equal loading control. Data from three independent experiments are presented. RTA wt is marked as 1, other results are relative to this control. **(C)** Coomasie Blue-stained 12% SDS/PAGE gels showing the stability of 500 ng of wild-type ricin, and modified ricin IHF and DHF after 30 min incubation at 0°C. **(D)** Cells were treated with or without bafilomycin A1 (0.1 μM) and with wild-type ricin, IHF ricin or DHF ricin. The amounts of ricin that remain in the cell after degradation was analyzed after SDS-PAGE run under non-reducing conditions (see Methods). Representative membranes after Western blot with anti-RTA antibodies are shown. Western blots with anti-tubulin antibodies were performed to show equal loading control. Data from three independent experiments are presented. Ricin wt, ricin IHF and ricin DHF in each graph are marked as 1, other results are relative to this control. All degraded forms of ricin were analyzed. **(E)** Tyrosine sulfated wild-type ricin sulf-1, and modified ricin sulf-1: IHF or DHF in cells after 3 hours incubation with Na_2_^35^SO_4_ and further 3 h incubation with the toxins, run on SDS/PAGE under non-reducing conditions. Control experiments revealed equal total sulfation of proteins in cell lysates. A representative autoradiogram is shown.

To investigate whether mutations in the hydrophobic region of RTA influence modified ricin A-chain cytotoxicity, HEK293 cells were incubated with increasing concentrations of wild-type or mutated forms (DHF, IHF) of ricin for 3 hours and the protein synthesis was then measured. Data show that the introduced mutations that changed hydrophobicity of the C-terminal region of RTA, completely abolished the cytotoxicity of ricin (Figure 3). To further explain this observation, we compared the intracellular amount of ricin DHF, ricin IHF and wild-type ricin. Cells were incubated with wild-type holotoxin or mutants with changed hydrophobicity for 3 hours. Then intact toxin was immunoprecipitated from the cell lysates, separated under non-reducing conditions and analyzed with anti-RTA antibodies. The results showed significant decrease in the amount of both ricin DHF and ricin IHF compared to the amount of wild-type toxin present in the cells (Figure 4A).

### Mutations that change hydrophobicity of RTA influence the binding of modified proteins to the cell surface as well as affect their endosomal-lysosomal degradation

Abolished cytotoxicity of ricin DHF and ricin IHF is at least partially connected with a decreased intracellular level of both proteins. To further investigate the reason for the reduced amounts of both mutated forms of ricin, measurement of binding of ricin holotoxin DHF and IHF to the cell surface was performed. Cells were incubated with ricin at 0°C. This temperature stabilizes ricin bound the cell surface [40]. In this experiment Western blot analysis with anti-RTA antibodies was applied (Figure 4B). In the case of both mutated forms of ricin (DHF and IHF), there was a significant decrease in the amount of cell surface-bound holotoxin proteins with changed hydrophobicity in comparison to the wild-type protein (Figure 4B). Observed differences were not due to the decreased stability of ricin DHF and ricin IHF after incubation on ice, as presented in Figure 4C. These data suggest surprisingly that introduced mutations in the hydrophobic region of RTA affect the binding of ricin IHF and ricin DHF to the cell surface. This may explain decreased intracellular levels of both forms of ricin (Figure 4A). There are not any published results describing the importance of RTA in the ricin holotoxin recognition and binding to the cell surface receptors. We can assume that interaction of modified ricin A-chains with the B-chain in the holotoxin is somehow altered, what might influence recognition of cell surface receptors by RTB (see Discussion).

To investigate whether decreased amounts of IHF ricin and DHF ricin might also result from the increased endosomal-lysosomal degradation of both mutated forms of ricin, the total amounts of wild-type, IHF ricin and DHF ricin were estimated by Western blot analysis in cells cultured with and without bafilomycin A1. Bafilomycin A1 is an inhibitor of the vacuolar H^+^-ATPase [41]. As shown in Figure 4D, bafilomycin A1 only slightly increases the intracellular amount of wild-type ricin but significantly elevates the amounts of the mutated forms of ricin. Importantly, the increase in the amounts of IHF ricin and DHF ricin in the presence of bafilomycin A1 is higher than for wild-type ricin when compared to their counterparts not treated with this inhibitor (Figure 4D). Therefore, both forms of ricin with increased and decreased hydrophobicity in the C-terminal region are more effectively degraded in low-pH compartments in comparison to the wild-type protein. Interestingly, bafilomycin A1 increases also the amounts of partly degraded forms of IHF holotoxin and DHF holotoxin. All this may explain decreased intracellular amount of ricin mutants.

It has been demonstrated that ricin carrying a point mutation in the hydrophobic region of RTA (P250A) is subjected to increased endosomal-lysosomal degradation and that mainly cathepsin B but also cathepsin D are responsible for the degradation of the ricin holotoxin P250A mutant in the cell [23]. In our next experiments we used pepstatin A, which is a potent inhibitor of aspartyl proteases, including cathepsin D, and CA074 methyl ester that blocks cysteine cathepsins, especially cathepsin B. We found that both cathepsins are also involved in the degradation of ricin IHF and ricin DHF (see Additional file 3).

### RTA mutants with changed hydrophobicity are transported to the Golgi apparatus and to the ER

To elucidate if IHF ricin and DHF ricin, similarly to the wild-type toxin, are transported to the Golgi apparatus, the amounts of wild-type and mutated forms of ricin sulfated in the Golgi complex were examined. For this purpose wild-type ricin sulf-1 as well as the mutants IHF ricin sulf-1 and DHF ricin sulf-1, modified ricin molecules containing a sulfation site in the A-chain, were used. When cells are incubated with ^35^SO_4_^2−^, the A-chain becomes radioactively labeled due to the sulfotransferase in the trans-Golgi cisternae [42], and the fate of the ^35^SO_4_^2−^-labeled ricin molecule can be studied. As shown in Figure 4E, sulfated IHF ricin holotoxin and DHF ricin holotoxin can be detected after 3 hours incubation with the toxin. Thus, despite a dramatic reduction in the amount of both types of ricin in the cell, small fractions are transported to the Golgi apparatus. This experiment also shows a detectable fraction of a sulfated RTA_DHF_ and RTA_IHF_. Since SDS-PAGE was performed under non-reducing conditions, it means that small amounts of mutated IHF holotoxin and DHF holotoxin reach the ER where they become reduced.

### Recognition of RTA_DHF_ by chaperone proteins EDEM1 and EDEM2 is significantly decreased

**Figure 5:**
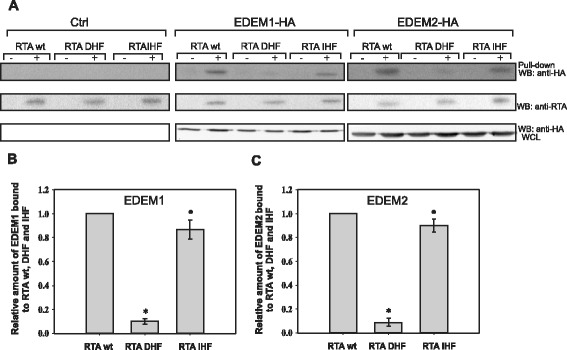
**Mutations that change hydrophobicity of the C-terminal region of ricin A-chain influence its interaction with EDEM1 and EDEM2. (A)** His-tag pull-down assay with RTA-His, RTA_DHF_-His and RTA_IHF_-His (0.5 μg each) and lysates from HEK293 cells overexpressing EDEM1-HA, EDEM2-HA or cells transfected with an control vector, Ctrl. Proteins bound to the Ni-NTA beads were analyzed by SDS/PAGE and Western blot with anti-HA antibodies (upper panel). The same membranes were re-probed with anti-RTA antibodies (middle panel). Whole cell lysates (WCL) analyzed with anti-HA antibodies are shown in the bottom panel. A representative experiment is shown. **(B)** Average data from His-tag pull-down with s.d. from four independent experiments. The amount of EDEM1 bound to RTA_DHF_ or RTA_IHF_ is relative to the amount of EDEM1 interacting with wild-type RTA which is marked as 1. Data were analyzed by Student’s *t*-test , *P < 0.001, ^•^ *P* = 0.112 **(C)** Average data from His-tag pull-down with s.d. from four independent experiments. The amount of EDEM2 bound to RTA_DHF_ or RTA_IHF_ is relative to the amount of EDEM2 interacting with wild-type RTA which is marked as 1. Data were analyzed by Student’s *t*-test, *P < 0.001, ^•^ *P* = 0.155.

It was shown previously that EDEM1 and EDEM2 proteins interact directly with the ricin A-chain [22-24]. The point mutation in the hydrophobic region of RTA (P250A) impairs interactions between EDEM1 and RTA [23] and between EDEM2 and RTA [24]. Since P250A mutation changes the conformation of the ricin A-chain into a more helical structure, it was concluded that the precise structure of RTA might determine its recognition by EDEM1 and EDEM2 [23,24]. To further investigate the mechanism of substrate recognition by EDEM1 and EDEM2, interaction of EDEM proteins with RTA_IHF_ and RTA_DHF_ was examined. We cloned and purified the RTA mutants with His-tag (see Methods) that were next used in the pull-down assays. In these experiments equal amounts of mutated forms of RTA and wild-type RTA were used. Figure 5 shows a significant decrease in RTA_DHF_ binding to EDEM1 and EDEM2 when compared to the wild-type RTA. Interactions between RTA_IHF_ and EDEM1 and between RTA_IHF_ and EDEM2 showed no statistically significant (p > 0.05) differences in comparison to interactions with wild-type RTA (Figure 5). These results indicate that for interactions between both EDEM1 and RTA and EDEM2 and RTA appropriate hydrophobicity of the substrate is important. In conclusion, reduced hydrophobicity of the C-terminal region of RTA results in impaired interactions with EDEMs chaperone proteins.

### Recognition of the C-terminal region of BACE457 by EDEM1 and EDEM2 depends on the hydrophobicity of this region

**Figure 6:**
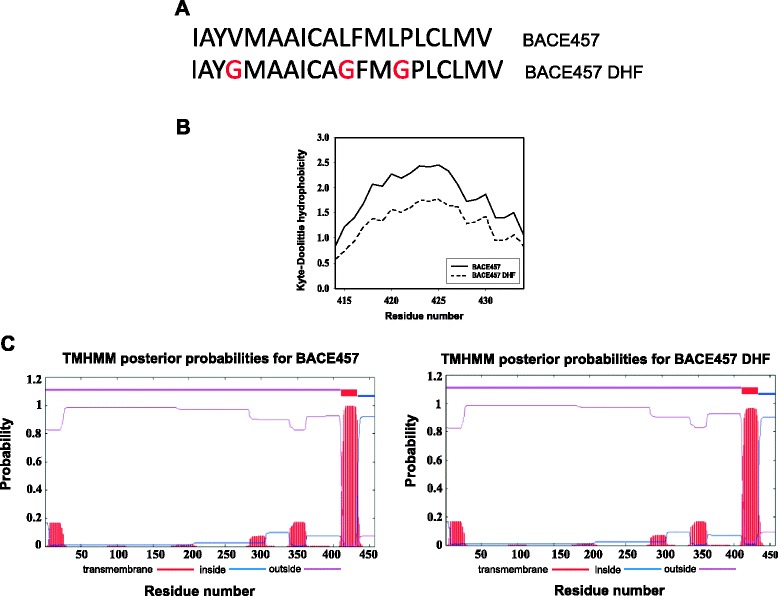
**Biochemical characteristics of BACE457**_**DHF**_ **. (A)** Sequence of the C-terminal hydrophobic region of BACE457. Introduced mutations are marked in red. **(B)** Hydrophobicity plot for the C-terminal region (Ile414-Val434) of BACE457 and BACE457_DHF_. Presented graph is a fragment of the output from ProtScale [64] prepared for full-length BACE457. A sliding window of 19 AAs was used. **(C)** Topology prediction of BACE457 and BACE457_DHF_ prepared by TMHMM Server v. 2.0 [66]. Graph shows the posterior probabilities of inside/outside/transmembrane helices. The red bars indicate predicted transmembrane regions, their length and posterior probabilities are indicated on X and Y axes respectively.

**Figure 7:**
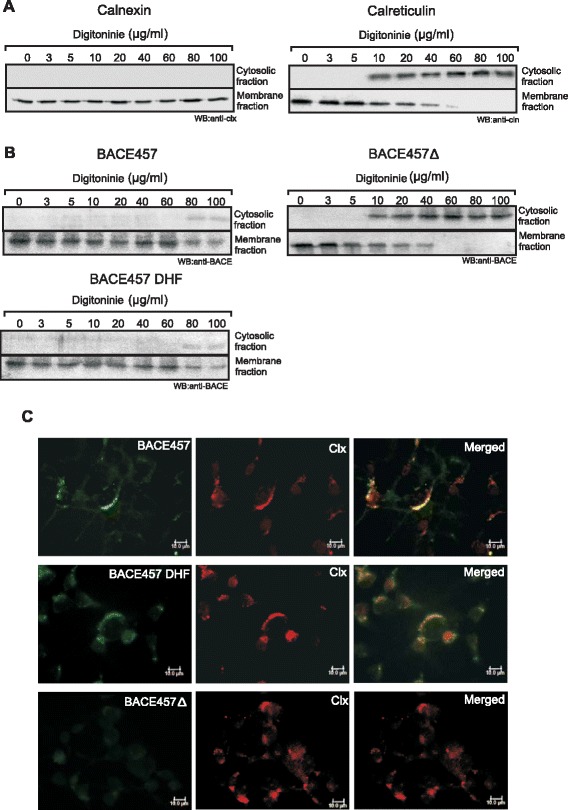
**BACE457**_**DHF**_ **is a membrane protein. (A)** Cells were subjected to permeabilisation (see Methods) with increasing concentrations of digitonin, concentrations as indicated. A representative membranes of cytosolic fraction and membrane fraction after Western blot with anti-calnexin (clx) or anti-calreticulin (cln) antibodies are shown. **(B)** Cells were transfected with cDNA encoding BACE457, BACE457_DHF_ or BACE457Δ and subjected to permeabilisation with increasing concentrations of digitonin, concentrations as indicated. A representative membranes of cytosolic fraction and membrane fraction after Western blot with anti-BACE antibodies are shown. **(C)** Cells were transfected with cDNA encoding BACE457, BACE457_DHF_ or BACE457Δ and subjected to permeabilisation with 60 μg/ml of digitonin. Then cells were fixed and stained as indicated in Methods. Bars, 10 μm.

To investigate whether the effect of the hydrophobicity is also important in interactions between EDEMs and a typical ERAD substrate, we used the model misfolded protein, BACE457. This protein is a spice variant of human brain isoform BACE501, β-site amyloid precursor protein cleaving enzyme, which is a type I transmembrane aspartyl protease. BACE457 is expressed in human pancreas, its gene lacks 132 base pairs, what causes an in-frame deletion of 44 amino acids in the full-length BACE501 [43]. It has been demonstrated that overexpression of EDEM1 accelerates degradation of BACE457 but has no effect on properly folded BACE501 [10]. Both forms of BACE have a hydrophobic region on their C-terminal end (in BACE457, Ile414-Val434) that serves as a transmembrane domain. It remains unclear how misfolded membrane proteins are selected and destroyed during ERAD, however, except mannose trimming from N-linked oligosaccharides, recently published results presented a possible role for the transmembrane regions in this process [44-46] (see also Discussion). To elucidate whether changed hydrophobicity of the C-terminal region of BACE457 would affect its interactions with EDEM1 and EDEM2 proteins, we cloned a mutated form of BACE457 (BACE457_DHF_), carrying three substitutions (V417G, L424G, L427G) (Figure 6A) that efficiently decrease hydrophobicity of the C-terminal region of this protein (Figure 6B). To minimalize the possibility of the disruption of the specific structure of this misfolded protein, we avoid any modifications of cysteine residues and did not modify any residues neighboring cysteines. *In silico* predictions revealed that these mutations did not change hydrophobicity to such an extent that it disturbs transmembrane location of this region (Figure 6C) or that it changes misfolding status (e.g. into more unfolded state) of the luminal part of BACE457 (see Additional file 4). Otherwise, these changes might influence ERAD [44-46] and affect interactions between BACE457 and EDEM proteins beyond the studied decreased hydropobicity of the C-terminal region of BACE457. Importantly, experimental data presented in Figure 7 confirms transmembrane localisation of modified BACE457_DHF_. Cells were permeabilized with a various concentrations (0–100 μg/ml) of a nonionic detergent, digitonin to separate cytosolic fraction form the membranes. Lower concentrations of digitonin, up to 5 μg/ml, selectively permeabilise plasma membrane, remaining ER membrane intact [22,23], higher concentrations of this detergent permeabilise also ER membrane causing leakage of soluble ER proteins to the cytosol, at a very high concentrations of digitonin the integrity of the ER membrane may be disturbed. Control experiments performed with a ER membrane protein, calnexin and a luminal protein calreticulin revealed stable transmembrane location of calnexin (Figure 7A). Calreticulin was observed in the membrane fraction as long as the ER membrane remained intact, at higher concentrations of digitonin, calreticulin was released to the cytosolic fraction (Figure 7A). Stable location of BACE457_DHF_ in the membrane fraction, similarly to BACE457, confirms ER membrane localization of BACE457_DHF_ (Figure 7B). In contrary to these observations, soluble form of BACE457 – BACE457Δ [47] was released to the cytosolic fraction at relatively low concentrations of digitonin (Figure 7B). To further confirm this observation, we investigated by confocal microscopy the localisation of BACE457, BACE457_DHF_ and BACE457Δ together with calnexin. Cells were permeabilised with 60 μg/ml of digitonin. Due to the Western blot analysis presented in Figures 7A, B such concentration of detergent leaves the ER membrane proteins in the membrane fraction and almost completely clears this fraction form the soluble ER proteins. BACE457 and BACE457_DHF_ were detected in the membrane fraction and both proteins were found to co-localise with calnexin (Figure 7C). BACE457Δ was not detected in this fraction what confirms our previous observations.

**Figure 8:**
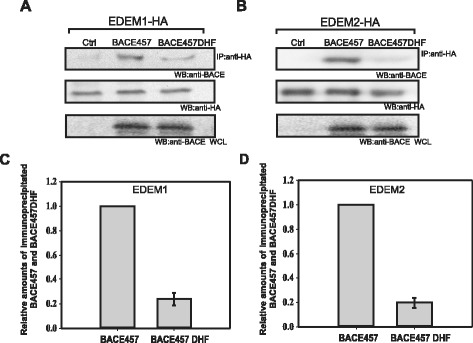
**Decreased hydrophobicity of BACE457 impairs its interactions with EDEM1 and EDEM2.** Co-immunoprecipitation of BACE457 or BACE457_DHF_ with anti-HA antibodies from cells overexpressing EDEM1-HA **(A)** or EDEM2-HA **(B)**. Representative examples of the experiments are shown. Membranes were re-probed with anti-HA antibodies to confirm equal immunoprecipitation. Whole cell lysates (WCL) analysed with anti-BACE antibodies are shown in the bottom panel. **(C)**, **(D)** Graphs of the average data presented in **(A)** and **(B)** with s.d. from three independent experiments are shown. The level of immunoprecipitated BACE457 from EDEM1-transfected cells or EDEM2- transfected cells is marked as 1, the level of immunoprecipitated BACE457 DHF is relative to this value.

To study interactions between BACE457_DHF_ and EDEM1 and between BACE457_DHF_ and EDEM2, we coimmunoprecipitated BACE457_DHF_ and in control experiments BACE457 with anti-HA antibodies from lysates of EDEM1-HA or EDEM2-HA-transfected cells (see Methods). As shown in Figure 8 BACE457 interacts with both EDEM1 and EDEM2. However, interactions of both chaperone proteins with BACE457_DHF_ are significantly reduced (Figure 8). It means that mutations responsible for decreased hydrophobicity of the C-terminal region of BACE457 influence its recognition by the chaperone proteins EDEM1 and EDEM2. These data indicate that EDEM proteins alone or as a part of larger complexes are able to recognize hydrophobic transmembrane domains of misfolded ERAD substrates. Additionally, it seems that sufficiently high hydrophobicity of protein substrate determinants is important for interactions with EDEM1 and EDEM2.

**Figure 9:**
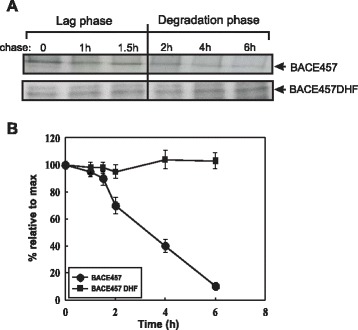
**Degradation of BACE457**_**DHF**_ **is completely inhibited. (A)** The amount of labeled BACE457 and BACE457_DHF_ remaining at the end of a chase, times as indicated. Representative examples of the experiments are shown. **(B)** Kinetics of degradation were determined by densitometric analysis of the labeled proteins. Graph of the average data with s.d. from three independent experiments is shown.

It is believed that EDEM1 and EDEM2 are crucial ER chaperons involved in ERAD disposition of aberrant glycoproteins [9-12]. To examine the degradation rate of BACE457_DHF_, HEK293 cells transfected with BACE457_DHF_ or BACE457 cDNAs were pulse-labeled with [^35^S]-Met, chased with unlabeled media up to 6 h followed by BACE457 immunoprecipitation from cell lysates. Degradation of BACE457 was started after a lag phase of about 90 min and then proceeded with a half-life of 3.5 hours (Figure 9). This is consistent with earlier published results [10,47]. Interestingly, degradation of BACE457_DHF_ was completely inhibited (Figure 9). We cannot exclude the possibility that high stability of changed BACE457 is at least partially connected with inhibited interactions with EDEM1 and EDEM2 proteins, however the possibility that decreased hydrophobicity of the C-terminal region of BACE457 results also in a lack of interactions with other important ERAD regulator(s) should be taken into consideration.

## Discussion

It is believed that mechanisms regulating recognition and degradation of folding-defective polypeptides expressed in the ER is one of the central issues determining the proper functioning of human cells. How proteins are sorted for ERAD is still poorly understood. Studies that have been performed during last decade show that EDEM family proteins are important regulators involved in disposal of misfolded glycoproteins from the ER [8-12,16-19].

In this study, we found that hydrophobic regions of the protein substrates seem to play a significant role in their recognition by the chaperone proteins EDEM1 and EDEM2. Recognition of ERAD substrates and protein toxins in the ER seems to be a complex issue. N-linked oligosaccharides are undoubtedly used as specific signal tags for ERAD and specific signals generated and/or recognized by EDEM proteins. EDEM proteins conserve all catalytic residues required for glycolytic activity and for binding of the specific inhibitor of α1,2 mannosidases, kifunensine [8,15,48]. It has been demonstrated that an increase of the intraluminal level of EDEM1 [17] and EDEM3 [18] substantially accelerates demannosylation of folding-defective polypeptides. Interestingly, it has been shown that at low EDEM1 levels its association with the ERAD substrate is glycan dependent whereas, at high levels it is not [49]. Glycan independent interactions of EDEM1 have been shown for: NHK, mutant variant of α1-antitrypsin (A1AT) [19], mutant P23H rod opsin [20] and the protein toxin ricin [22,23]. In the case of ricin, interactions with EDEM2 were also not dependent on carbohydrates [24]. Moreover, it has recently been shown that EDEM1 can associate with a glycoprotein substrate in the absence of the mannose-trimming activity [50]. All these observations led to the suggestion that in addition to N-linked oligosaccharide moieties of glycoproteins, EDEM proteins can recognize misfolded regions of aberrant proteins.

Ricin is not a typical ERAD substrate, however after holotoxin reduction in the ER, ricin A-chain utilizes ERAD and its components in its retrotranslocation to the cytosol [22-24,30,32]. It has been demonstrated previously that a point mutation (P250A) in the hydrophobic region of RTA alters the secondary structure of RTA into a more helical structure without affecting the hydrophobicity of the C-terminal region of RTA [23]. The interactions between EDEM1 and RTA_P250A_ [23] and EDEM2 and RTA_P250A_ [24] were significantly reduced indicating that EDEM1 and EDEM2 protein recognition might be determined by the structure of the ERAD substrate.

In this study we have cloned genes coding for ricin A-chain mutants with decreased (DHF) and increased (IHF) hydrophobicity of the C-terminal region of RTA, to further characterize the molecular basis of substrate recognition by EDEM1 and EDEM2. Binding of both mutated forms of ricin to the cell surface receptors was significantly decreased. In ricin holotoxin, the B- chain is a lectin, which binds to β-1,4-linked galactose residues widely present on mammalian cell surface glycoproteins and glycolipids. There is no direct contribution of ricin A-chain to the recognition and receptor binding of the ricin holotoxin. Re-association of RTA_DHF_ or RTA_IHF_ with RTB produces a holotoxin with a reducible disulfide bond. However, due to the significant hydrophobic changes in the mutated RTA (that might result in induction of an atypical entropic and enthalpic penalties of hydratation [51]), modified holotoxins can have altered conformation in comparison to the wild-type holotoxin e.g. changed localization of RTB in relation to RTA. This might influence binding of mutated forms of ricin holotoxin to the cell surface receptors. Decreased degradation of ricin DHF and ricin IHF in the presence of inhibitors of aspartyl and cysteine proteases represents the ricin transported to endosomes and lysosomes in an ER-independent pathway. However, we cannot exclude the possibility that the ricin mutated forms can also be affected by UPS (ubiquitin proteasome system)-mediated degradation. Despite decreased intracellular levels of ricin DHF and ricin IHF, both forms reached the Golgi complex and then the ER. However, due to the small amounts of mutated ricin A-chains present in the ER, it was impossible to study their retrotranslocation to the cytosol and examine their sensibility to proteasomal degradation, as was done for RTA_P250A_ [23,24]. Importantly, pull-down experiments revealed that EDEM1 and EDEM2 recognize ricin A-chain with decreased hydrophobicity with significantly lower affinity. Thus, both EDEM proteins bind exposed hydrophobic domains that possess appropriate high hydrophobicity. On the other hand, increased hydrophobicity of the C-terminal region of ricin A-chain does not result in elevated level of binding to EDEM1 and EDEM2. We cannot however exclude the possibility that other chaperone protein(s) present in the cell lysate bind the mutated highly hydrophobic region of RTA so strongly that they inhibit to some extent RTA_IHF_ interactions with EDEM1 and EDEM2. Alternatively, we can speculate that in the case of RTA_IHF_ the increase in the hydrophobicity was not high enough to observe increased level of binding to EDEM1 and EDEM2.

BACE457 is relatively well characterized ERAD substrate. Most of the proteins transiently expressed in human cells undergo inefficient folding in the ER, leading to proteasomal degradation. Detailed analysis revealed that BACE457 is firstly directed to the ER folding machinery, the calnexin cycle. After an insufficient folding process, extensively oxidized BACE457 transiently enters in disulfide-bonded complexes associated with the luminal chaperone BiP and protein disulfide isomerase (PDI) [47]. EDEM1 overexpression resulted in faster release of BACE457 from the calnexin cycle and earlier onset of its degradation, whereas EDEM1 downregulation prolonged its folding attempts and delayed ERAD [10]. Moreover, it has been demonstrated, that elevation of the intralumenal concentration of EDEM1 accelerated ERAD of BACE457 by efficient de-mannosylation of N-linked oligosaccharides [17]. In this study, we show that EDEM1 and EDEM2 are able to recognize the hydrophobic region of BACE457 which may significantly contribute to its degradation, since BACE457 with a mutation that decreases hydrophobicity of this region is less effectively recognized by EDEM1 and EDEM2, and degradation of BACE457_DHF_ is abolished. These results are in agreement with data for RTA_DHF_, confirming that EDEM1 and EDEM2 recognize protein determinants possessing appropriately high hydrophobicity. This recognition might represent an important step in the differentiation between proteins undergoing a folding process and terminally misfolded proteins directed for degradation. The differentiation might be based on more extensive exposure of hydrophobic domains by terminally misfolded glycoproteins.

Moreover, these data contribute to our overall understanding of ERAD of membrane proteins. Integral membrane substrates expose one or more regions to the cytoplasm, which can be ubiquitinated. Under debate is whether ubiquitinated membrane proteins are degraded by the proteasome in situ at the membrane or whether they are extracted prior to proteolysis. Alternatively, degradation could start from an internal site on a cytoplasmically exposed loop after an endoproteolytic clip by the proteasome [45]. In these models, it is assumed that degradation and retrotranslocation are tightly coupled and occur at the ER membrane. On the other hand, some integral membrane ERAD substrates, such as MHCI [52] and cystic fibrosis transmembrane conductance regulator (CFTR) [53], have been observed to reside in the cytoplasm when proteasome function is compromised. These data suggested that transmembrane hydrophobic segments might be solubilized from the lipid bilayer of the ER prior to proteasome-mediated degradation. Indeed, it has been demonstrated that the transmembrane domain of the ERAD substrate, Ste6p, is released into the cytosol in a Cdc48/p97- and ATP-dependent manner [44]. Candidates for factors that could maintain the solubility of transmembrane domains include cytoplasmic chaperones (such as Cdc48p), proteasome associated factors such as Rad23p/Dsk2p [54], and the 19S particle. Interestingly, it has been recently demonstrated that the proteasome 19S subunit can act as chaperone protein also for ricin A-chain, preventing aggregation of unfolded RTA [55]. Moreover, it was suggested that misfolding status of the cytoplasmatic domains of membrane ERAD substrates might influence the assembly of the intramembrane domain, which is recognized by Hrd1p, an E3 ubiquitin ligase complex [44]. Some reports suggest that an Hsp70 family member-containing or putative lectin-containing complex in the ER helps to recruit a misfolded substrate to the Hrd1p complex [56-58]. The mannosidase-like domain of EDEM1 does not appear to be required for ERAD substrate binding, but this domain is involved in binding to SEL1L-containing ER membrane dislocation and ubiquitination complex [19]. It is possible that this domain also recognizes hydrophobic patches of aberrant proteins. We cannot exclude the possibility that recognition of hydrophobic transmembrane domains by EDEM1 and EDEM2 is necessary for solubilization from the lipid bilayer or that EDEMs recognize already solubilized transmembrane domains. Importantly, it has been demonstrated that several less-hydrophobic transmembrane sequences derived from multimeric transmembrane protein complexes can enter the ER lumen completely, where they are recognized as substrates of the chaperone BiP, which in turn initiate degradation of the unassembled subunit [59]. This new mechanism suggests a general link between proper integration of transmembrane segments and the ER luminal chaperone machinery. Interactions between hydrophobic transmembrane domain of BACE457 and EDEMs might explain differences between ERAD of membrane BACE457 and its luminal form BACE457∆. Degradation of BACE457∆ is faster than that of membrane BACE457; half-life of BACE457∆ is about 40 min, while that of BACE457 is 4 h [47]. Importantly, the lag phase for the soluble variant of BACE457 is only 15 min, whereas for membrane bound BACE457 it is 90 min [47]. These differences might be connected with necessity of extraction of transmembrane domain of BACE457 out of the ER membrane. Hebert and colleagues suggested that EDEM1 serves as a quality control receptor that acts as a molecular link between misfolded proteins and SEL1L [19]. Recently published results show that degradation of BACE457 did not require SEL1L complex [60]. However, ricin A-chain transport to the cytosol depends on SEL1L [32]. It is possible that the role of EDEM1 and EDEM2 in ERAD in some aspects is common for luminal and membrane substrates: extraction from the calnexin/calreticulin cycle and substrate de-mannosylation (at least in the case of EDEM1). However, after release from EDEM1 or/and EDEM2, terminal acceptors of misfolded membrane proteins might be different from luminal aberrant glycoproteins.

About one-third of all mammalian genes encode secreted and membrane proteins, including numerous important molecules such as ion channels and cell surface receptors [61]. The high rate of protein synthesis and the large fraction of misfolded and unassembled proteins generated in the ER, indicate that the ERAD plays a central role during active secretion, cell growth, and normal turnover in eukaryotic cells. Understanding the mechanism of ER protein quality control, specifically of how the cell recognizes and discriminates misfolded glycoproteins remains to be one of the central issues in cell biology.

## Conclusions

We have demonstrated that ricin A-chain and a model misfolded protein, BACE457 carrying mutations that significantly decreased their hydrophobicity, are poorly recognized by EDEM1 and EDEM2 in contrast to their wild-type counterparts. Moreover, degradation of mutated BACE457 with decreased hydrophobicity is significantly reduced. The data presented in this paper contribute to the general understanding of the mechanism of recognition of misfolded proteins in the ER and the role of EDEM1 and EDEM2 chaperone proteins in this process.

## Methods

### Materials

Ricin B-chain was obtained from Vector Labs (Burlingame, CA), HEPES, α-lactose monohydrate, trypsin, pronase, bafilomycin A1, pepstatin A and CA074 methyl ester came from Sigma-Aldrich (St. Louis, MO). [^3^H]Leucine was purchased from GE Healthcare (Princeton, NJ), Na_2_^35^SO_4_ and L-[^35^S]Methionine came from Hartmann Analytic (Braunschweig, Germany). The mouse monoclonal anti-HA antibodies were obtained from Covance Research Products (Denver, CO), rabbit anti-BACE from Merck (Whitehouse Station, NJ), rabbit anti-Ricinus Communis-Lectin, and mouse anti-α-tubulin came from Sigma-Aldrich, whereas mouse monoclonal anti-ricin A-chain were purchased from Serotec (Oxford, UK). The mouse anti-calnexin were obtained from BD Biosciences (Palo Alto, CA), anti-calreticulin from BioSite (San Diego, CA). The rabbit anti-His antibodies came from Santa Cruz Biotechnology (Santa Cruz, CA). The secondary anti-rabbit HRP and anti-mouse HRP antibodies, were obtained from Sigma-Aldrich, whereas anti-rabbit Alexa555 and anti-mouse Cy-3 were obtained from Jackson laboratories (Bar Harbour, MA).

### Cell culture

Human Embryonic Kidney 293 (HEK293) cells were grown under 5% CO_2_ in DMEM (Sigma-Aldrich) supplemented with 10% fetal bovine serum (FBS), 25 U/ml penicillin, and 25 μg/ml streptomycin (all from Sigma-Aldrich) in a 37°C incubator.

### Cloning, mutagenesis, cDNA constructs and transfections

The ricin A-chain mutants with decreased hydrophobicity (RTA_DHF_) and increased hydrophobicity (RTA_IHF_) were obtained by site-directed mutagenesis. The following primers were used: for RTA_DHF_ 5’-GTGTGTACGATTCGAGTATAAATATCCCTATCAATTCGCTCATGGTGTATAG- 3’ and 5’-CTATACACCATGAGCGAATTGATAGGGATATTTATACTCGAATCGTACCAC-3’ and for RTA_IHF_ 5’-GTGTGTACGATGTGGTTATATTAATCCCTATCATAGTACTCATGGTGTATAG-3’and 5’- CTATACACCATGAGTACTATGATAGGGATTAATATAACCACATCGTACACAC- 3’ The ricin A-chain sulf-1 cDNA [62] or ricin A-chain fused to 6XHis [23] were used as templates and the reactions were performed by the standard procedure [63]. BACE457 with decreased hydrophobicity (BACE457_DHF_) was obtained by site direct mutagenesis using primers: 5’-GATGAGTCAACCGGCATG ACCGGAGCCTATGGCATGGCTGCCATCTGCGCCGGCTTCATGGGGCCACTCTGC-3’and 5’-GCAGAGTGGCCCCATGAAGCCGGCGCAGATGGCAGCCATGCCATAGG CTCCGGTCATGCCGGTTGACTCATC-3’. BACE457 cDNA (a kind gift from Dr. Paolo Paganetti, Novartis Pharma AG, Basel, Switzerland) [10] was used as a template. Dr. Paolo Paganetti provided also cDNA encoding soluble form of BACE457 – BACE457∆.

cDNA encoding the mouse EDEM1 fused to an hemagglutinin (HA) tag in the pCMV-SPORT2 vector was a kind gift from Prof. Kazuhiro Nagata and Prof. Nobuko Hosokawa (Institute for Frontier Medical Sciences, Kyoto University, Japan). For details of the cloning refer to [8]. cDNA encoding the mouse EDEM2 fused to an hemagglutinin (HA) tag in the pRK7 vector was a generous gift from Prof. Maurizio Molinari (Institute for Research in Biomedicine, Basel, Switzerland). For details of the cloning refer to [12].

HEK293 cells were transiently transfected with FuGENE™ transfection reagent (Roche Diagnostics, Basel, Switzerland) according to the manufacturer’s procedure or TurboFect (Life Technologies, Carlsbad, CA).

### Purification of ricin A-chain proteins and reconstitution with ricin B-chain

RTA His-tag, modified RTA_DHF_ His-tag and RTA_IHF_ His-tag were expressed in *E. coli* Rosetta cells (Merck) and purified using Ni-NTA agarose beads (Qiagen, Germantown, MD) according to the manufacturer’s manual. The eluate was finally dialysed overnight in PBS.

Ricin A-chain sulf-1 and modified RTA_IHF_ and RTA_DHF_ sulf-1 fused to maltose binding protein (MBP) were applied to a column with amylose resin and purified as previously described [22]. Free wild-type RTA, RTA_DHF_ or RTA_IHF_ were cleaved off with factor Xa (New England Biolabs, Ipswich, MA). For further purification, wild-type RTA, RTA_DHF_ or RTA_IHF_ proteins were applied on a MonoS column (GE Healthcare) and purified using GE Pharmacia Acta Purifier (GE Healthcare). 25 mM phosphate buffer, pH 6.5 was used as the column equilibrating buffer and the wash buffer, proteins were eluted with 0–500 mM NaCl gradient, and the fractions containing ricin A-chain were identified by Coomassie-stained SDS/PAGE. Purified wild-type RTA, RTA_DHF_ or RTA_IHF_ were mixed with the ricin B-chain and dialyzed extensively against PBS to remove reducing agents.

### Protease digestion assays

500 ng of RTA, RTA_DHF_ or RTA_IHF_ were incubated with increasing concentrations of trypsin (0–100 μg/ml) in NaCl/P_i_ (137 mM NaCl, 2.7 mM KCl, 10 mM Na_2_HPO_4_, 1.8 mM KH_2_PO_4_, pH 7.4) at 37°C for 15 min and then visualized by SDS/PAGE and Coomassie Blue staining. Pronase digestion was performed at 40°C for 20 min in a buffer containing 0.1 M Tris–HCl, pH 6.5 and 0.5% SDS with increasing concentrations of the protease (0–1.5 μg/ml), inactivation of the protease was done at 80°C for 5 min. Proteins were visualized by SDS/PAGE and Coomassie Blue staining. Denatured forms of RTA were prepared by incubation of RTA at 85°C for 30 min in 25 mM phosphate buffer, pH 6.5.

### The interchain disulfide bond stability

100 ng reassociated wild-type RTA:RTB, RTA_DHF_:RTB or RTA_IHF_:RTB were incubated with increasing concentrations (0.1-10 mM) of dithiothreitol (DTT) at 37°C for 30 min. The reaction was quenched with iodoacetamide (final concentration 20 mM) at 37°C for 10 min and then visualized by SDS/PAGE and Coomassie Blue staining. Denatured forms of ricin were prepared by incubation of RTA at 85°C for 30 min in 25 mM phosphate buffer, pH 6.5.

### Circular dichroism

Far-UV CD was measured on Jasco J-815 spectrapolarimeter (Jasco, Tokyo, Japan). Experiments were performed in 25 mM phosphate buffer, pH 6.5 using a 1-mm-path length cuvette. The concentration of the protein solutions were 30 μg/ml. Spectra recorded from 190 to 240 nm with 1-nm step size were averaged from three accumulations and were corrected against the buffer.

### Measurements of protein synthesis

HEK293 cells were washed in leucine free Hepes-buffered medium and incubated with the same type of medium with different concentrations of wild-type or mutant ricin DHF or IHF for 3 hours. The cells were then incubated in leucine free medium supplemented with 1 μCi/ml [^3^H]leucine for 20 minutes at 37°C. Cells were extracted with 5% trichloroacetic acid (TCA) for 20 minutes, followed by a wash (5 minutes) in 5% TCA and subsequently dissolved in 0.1 M KOH. The cell-associated radioactivity was measured. The results are expressed in percent of [^3^H]leucine incorporated in cells incubated without toxin. Deviations between duplicates did not vary by more than 10%.

### Measurements of binding and degradation of wild-type ricin, IHF ricin and DHF ricin

Cell surface binding of wild-type ricin, IHF ricin and DHF ricin was measured after 30 min incubation with ricin (~100 ng/ml) at 0°C in Hepes medium. After incubation, cells were washed four times with ice-cold Hepes-buffered medium. For the wild-type ricin, IHF ricin and DHF ricin visualization, Western blot with anti-RTA antibodies was employed. Stability of all types of ricin was verified in a separate control experiment after 30 min incubation on ice. Proteins were visualized by SDS/PAGE and Coomassie Blue staining. For degradation measurement, cells were treated either with bafilomycin A1, pepstatin A, CA074 methyl ester, or a combination of pepstatin A and CA074 methyl ester, for 30 minutes, and then incubated with wild-type ricin, IHF ricin or DHF ricin for 3 hours. To determine the total amount of ricin remaining in the cells after degradation, Western blot with anti-RTA antibodies was performed. Concentrations of inhibitors are indicated in the figure legends (Figure 4 and see Additional file 3).

### Sulfation of wild-type ricin sulf-1 and mutants with increased or decreased hydrophobicity

HEK293 cells were incubated with approximately 500 ng/ml of wild-type, DHF ricin or IHF ricin sulf-1 in Hepes medium for 3 hours at 37°C. For the sulfated ricin analysis, cells were incubated with 0.2 mCi/ml Na_2_^35^SO_4_ in DMEM without sulfate for 3 hours before toxin was added, and the incubation was continued for the next 3 hours at 37°C. Cells were then incubated twice (5 min) in a 0.1 M lactose solution at 37°C, to remove surface bound toxin, then washed once with cold PBS, and lysed (lysis buffer: 0.1 M NaCl, 10 mM Na_2_HPO_4_, 1 mM EDTA, 1% Triton X-100, pH 7.4) in the presence of a protease inhibitor mixture (Roche Diagnostics). Wild-type ricin, IHF ricin or DHF ricin were immunoprecipitated from the fractions with rabbit anti-ricin antibodies and immobilized on protein A-Sepharose CL-4B (GE Healthcare). Finally, the beads were washed with cold PBS supplemented with 0.35% Triton X-100, and the adsorbed material was analyzed by SDS-PAGE (12%) under non reducing conditions. For the detection of ^35^SO_4_^2−^-labeled ricin, dried membranes were exposed to Kodak BioMax MR film (Kodak, Rochester, NY) at room temperature. The radioactivity in the cell lysates was measured to detect possible differences in the total amount of isotope incorporated under different conditions. For total ricin analysis in the cell, Western blot analysis with anti-RTA antibodies was performed. Holotoxin was detected by chemiluminescence reagent SuperSignal WestPico Chemiluminescent Substrate (Thermo Scientific, Waltham, MA). Signal intensities of the bands were quantified using ImageQuant 5.0 software (GE Healthcare).

### Permeabilisation of cells

HEK293 cells were washed once with PBS and incubated with increasing concentrations of digitonin (0–100 μg/ml) for 30 min as previously described [22]. After collection of cytosolic fraction, the cells were lysed as described above. The supernatant (containing cytosolic fraction) and lysates (obtained from the remaining membrane fraction) were centrifuged to remove cell debris and nuclei for 10 min at 10’000 g. Western blots with anti-calnexin, anti-calreticulin or anti-BACE antibodies and appropriate secondary antibodies were performed. Proteins were detected by chemiluminescence reagent SuperSignal WestPico Chemiluminescent Substrate (Thermo Scientific).

### Immunofluorescence microscopy

HEK293 cells transfected with cDNA encoding BACE457_,_ BACE457_DHF_ or BACE457Δ were grown on coverslips. The cells were then subjected to permeabilisation with 60 μg/ml of digitonin (30 min), washed once with PBS, and fixed in 3% paraformaldehyde (PFA, Sigma-Aldrich). Cells were then permeabilised in 0.1% Triton X-100 and blocked in 5% FCS before labelling with rabbit anti-BACE together with mouse anti-calnexin and treated with the appropriate secondary antibodies. The cells were mounted in Mowiol (Life Technologies) and examined with a Leica DMI4000B automated inverted microscope.

### His-tag pull-down assay

HEK293 cells (1×10^5^/plate) were seeded in 6 cm plates and transfected with either EDEM1-HA, EDEM2-HA cDNA or a control vector. Three days post transfection cells were lysed (lysis buffer: 50 mM Hepes, pH 7.5; 150 mM NaCl; 10% glycerol and 1% Triton X-100) in the presence of a protease inhibitor mixture (Roche Diagnostics) and sonicated (5’, 20% output). His-tag fusion proteins: wild-type RTA or mutant RTA_IHF_, RTA_DHF_ (0.5 μg) were incubated at 37°C for 2 h with lysates from cells transfected with the indicated constructs and then incubated with Ni-NTA agarose beads (Qiagen). Beads were washed three times with lysis buffer supplemented with 30 mM imidazole and resuspended into a SDS-PAGE sample buffer. Amounts of toxin-bound EDEM1-HA or EDEM2-HA were detected after Western blot with anti-HA antibodies. Signal intensities of the bands were quantified using ImageQuant 5.0 software (GE Healthcare). Membranes were re-probed with anti-RTA antibodies to confirm equal amounts of RTA-His, RTA_DHF_-His and RTA_IHF_-His used in the experiments.

### Metabolic labeling of BACE457 and immunoprecipitation

HEK293 cells (1×10^5^/plate) were seeded in 6 cm plates and transfected with either BACE457 cDNA or BACE457_DHF_ cDNA. Three days post transfection cells were starved for 30 min in methionine free MEM medium (Sigma-Aldrich), pulsed for 10 min with 150 μCi [^35^S]methionine and chased for 0, 1, 1.5, 2, 4, 6 hours in complete medium supplemented with 5 mM methionine. Then cells were lysed in Hepes buffered saline (HBS), pH 6.8 containing 2% CHAPS and a protease inhibitor mixture (Roche Diagnostics). Cell lysates were prepared by centrifugation at 10,000 *g*, 10 min to remove cell debris and nuclei. For immunoprecipitation of BACE457 or BACE457_DHF_, lysates were incubated 4 hours at 4°C with protein A-Sepharose CL-4B (GE Healthcare) precoated with anti-BACE antibodies. Finally, the beads were washed three times with cold HBS buffer supplemented with 0.5% CHAPS and 0.1% Tween 20, and the adsorbed material was analyzed by SDS-PAGE (12%) under reducing conditions. Densitometric quantification of BACE457 bands was performed using ImageQuant 5.0 software (GE Healthcare). Percentage of degradation was determined in at least four independent experiments.

### Softwares and bioinformatic servers used for protein characterization

ProtScale [64] was used for generating hydrophobicity plots with Kyte-Doolittle [65] hydrophobicity scale. Topology prediction of BACE457 and BACE457_DHF_ were prepared by TMHMM Server v. 2.0 [66]. Predicted models of proteins were prepared by Geno3D server [39]. Proteins were visualized in the PyMOL Molecular Graphics System, Version 1.3. Schrödinger, LLC [67].

### Statistics

All experiments were performed independently at least three times. Values of 3 or more parallels were given as a mean ± standard deviation (s.d.). A *P* value of 0.05 or less was considered to be statistically significant and determined by the Student’s *t*-test or ANOVA tests.

## Additional files

Additional file 1:
**Characteristics of RTA**_**DHF**_ **and RTA**_**IHF**_ **.**
Additional file 2:
**Stability of RTA**_**DHF**_ **and RTA**_**IHF**_ **.**
Additional file 3:
**DHF ricin and IHF ricin show increased degradation in low pH vesicles.**
Additional file 4:
**BACE457 and BACE457**_**DHF**_ **luminal parts predicted ribbon models.**

## Abbreviations

ER: Endoplasmic reticulum
RTA: Ricin A-chain
RTB: Ricin B-chain
P250A: Substitution of proline 250 into alanine
RTA_DHF_: Ricin A-chain with decreased hydrophobicity in the C-terminal region
RTA_IHF_: Ricin A-chain with increased hydrophobicity in the C-terminal region
EDEM1: ER degradation enhancing α-mannosidase I-like protein 1
EDEM2: ER degradation enhancing α-mannosidase I-like protein 2
ERAD: ER-associated protein degradation
TCA: Trichloroacetic acid
CHAPS: 3-[(3-cholamidopropyl)dimethylammonio]propane-1-sulfonic acid, HA- hemagglutinin
