# Supplementary material for: Hydrophobicity of protein determinants influences the recognition of substrates by EDEM1 and EDEM2 in human cells
Source: BMC Cell Biol. 2015 Feb 6;16:1. doi: 10.1186/s12860-015-0047-7 (PMC4340280; doi:10.1186/s12860-015-0047-7)

**Additional file 1. Characteristics of RTA$_{DHF}$ and RTA$_{IHF}$. (A)** Wild-type RTA, RTA$_{DHF}$ and RTA$_{IHF}$ predicted ribbon models. Models were prepared by Geno3D server [36], proteins were visualized in the PyMOL [64]. The C-terminal hydrophobic region (Val245-Val256) of ricin A-chain in all models is shown in green. **(B)** Combined fragments of the backbone models of wild-type ricin A-chain (marked in red, hydrophobic region in green), DHF ricin A-chain (marked in turquoise) and IHF ricin A-chain (marked in pink). In the foreground hydrophobic regions are presented, sticks are indicated. **(C)** Electrostatic surface models for structures presented in (A). Color code is indicated (local PyMOL scale).

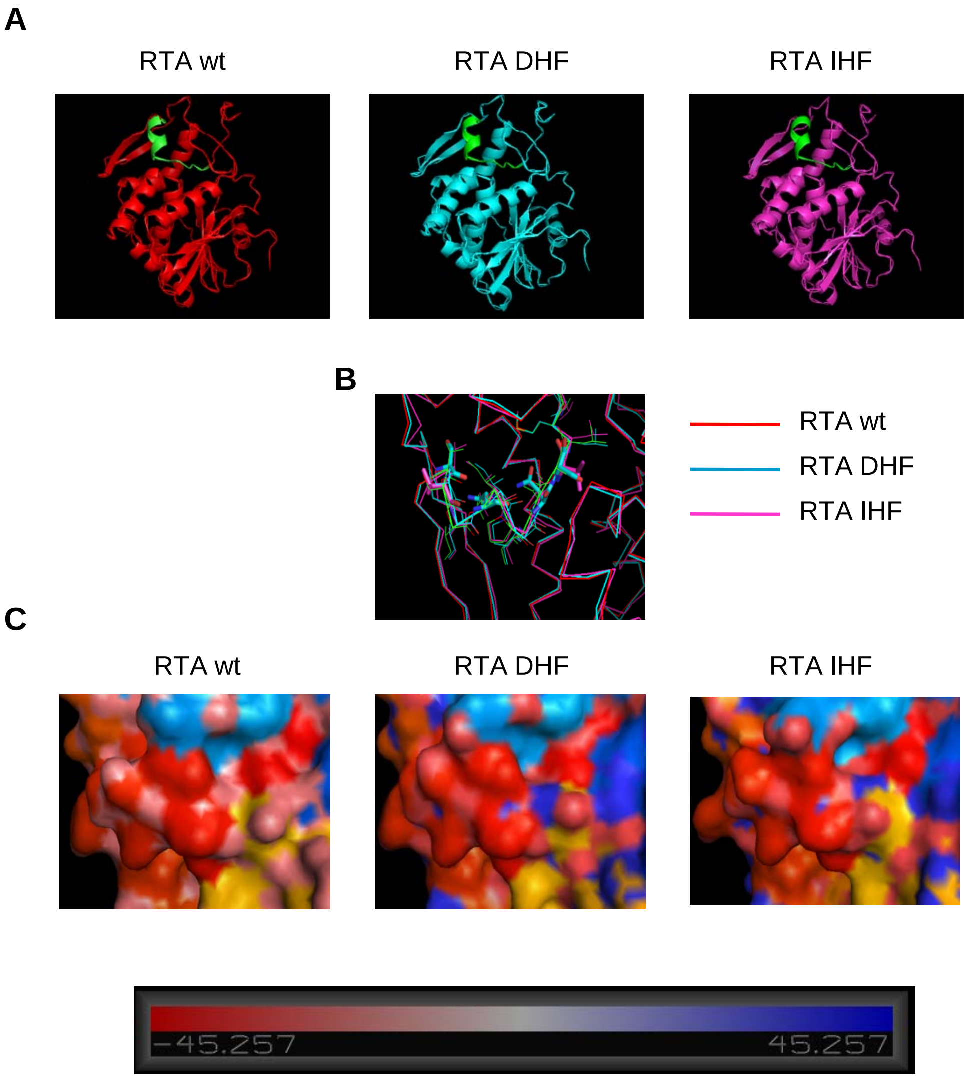

Supplement: Additional file 1: — Characteristics of RTA DHF and RTA IHF . [file 12860_2015_47_MOESM1_ESM.pdf]
