# Supplementary material for: Hydrophobicity of protein determinants influences the recognition of substrates by EDEM1 and EDEM2 in human cells
Source: BMC Cell Biol. 2015 Feb 6;16:1. doi: 10.1186/s12860-015-0047-7 (PMC4340280; doi:10.1186/s12860-015-0047-7)

Additional file 2. Stability of RTA$_{DHF}$ and RTA$_{IHF}$. Coomasie Blue-stained 12% SDS/PAGE gels showing the effect of the incubation of 500 ng of wild-type ricin A-chain, RTA wt, and modified ricin A-chains: RTA$_{DHF}$ and RTA$_{IHF}$, with increasing concentrations of pronase. Digestion patterns for denatured forms of RTA are also shown.

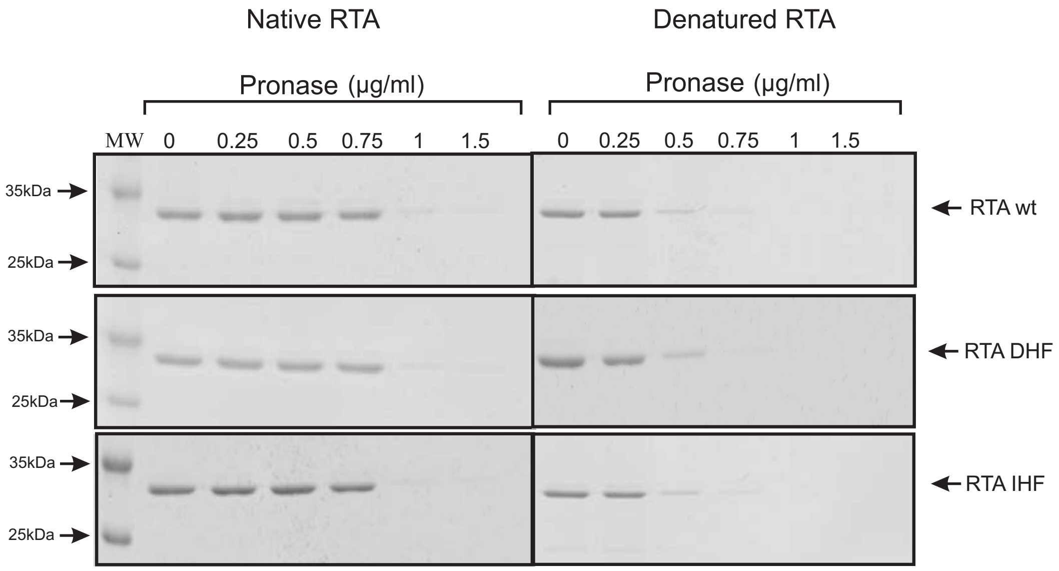

Supplement: Additional file 2: — Stability of RTA DHF and RTA IHF . [file 12860_2015_47_MOESM2_ESM.pdf]
