# Supplementary material for: Hydrophobicity of protein determinants influences the recognition of substrates by EDEM1 and EDEM2 in human cells
Source: BMC Cell Biol. 2015 Feb 6;16:1. doi: 10.1186/s12860-015-0047-7 (PMC4340280; doi:10.1186/s12860-015-0047-7)

Additional file 3. DHF ricin and IHF ricin show increased degradation in low pH vesicles. HEK293 cells were treated with or without pepstatinA(10 μg/ml)**(A)**; CA074 methyl ester (CA074-Me) (50 μM) **(B);** or a combination of pepstatinA and CA074 methyl ester (inhibitors) **(C)** and with wild-type ricin, IHF ricin or DHF ricin. The amounts of ricin that remain in the cell after degradationwas analyzed after SDS-PAGE run under non-reducing conditions (see Methods). Representative membranes after Western blot with anti-RTA antibodies are shown.Western blots with anti-tubulin antibodies were performed to show equal loading control. Data from three independent experiments are presented. Ricin wt, ricin IHF and ricin DHF in each graph are marked as 1, other results are relative to this control. All degraded forms of ricin were analyzed.

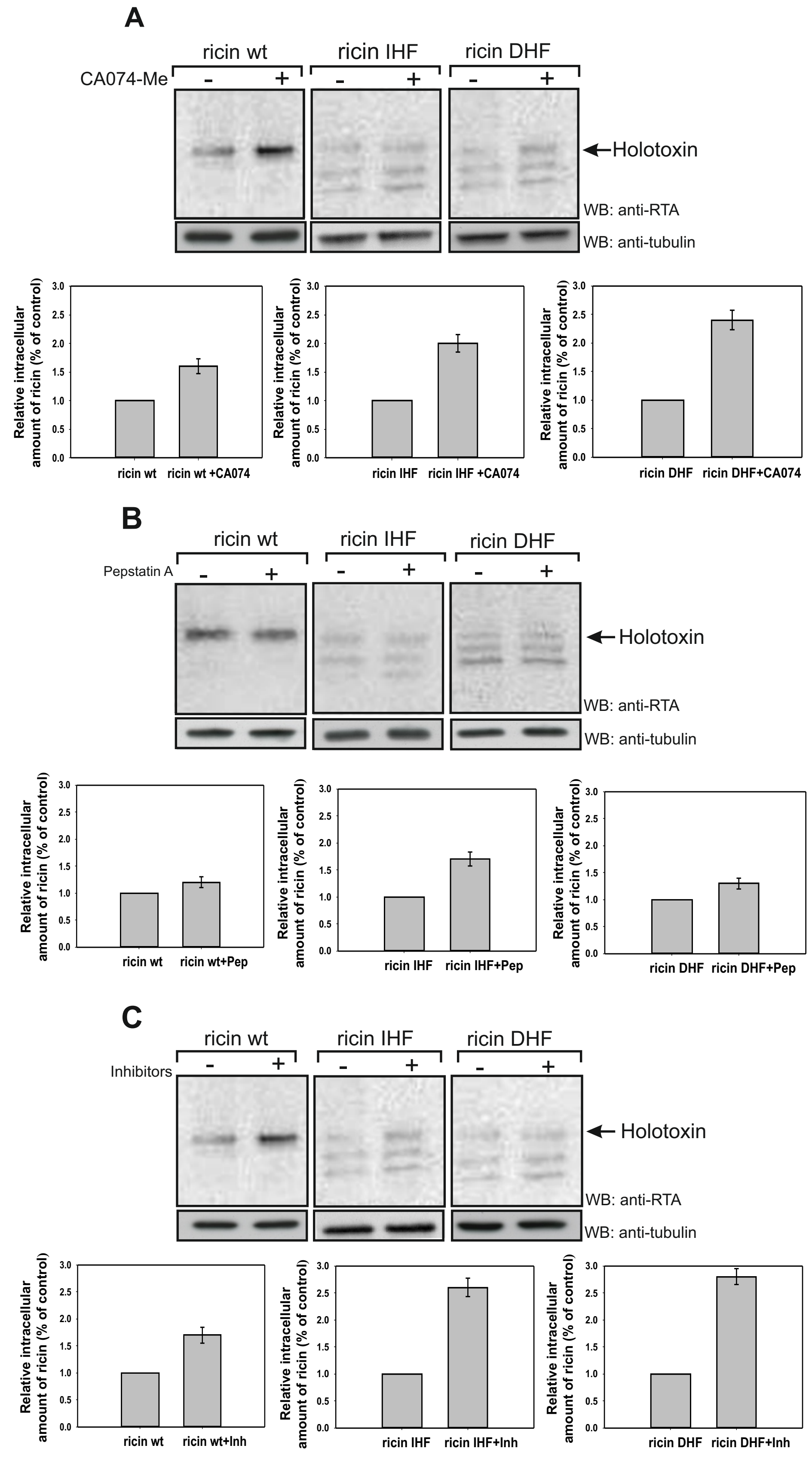

Supplement: Additional file 3: — DHF ricin and IHF ricin show increased degradation in low pH vesicles. [file 12860_2015_47_MOESM3_ESM.pdf]
