# Supplementary material for: Hydrophobicity of protein determinants influences the recognition of substrates by EDEM1 and EDEM2 in human cells
Source: BMC Cell Biol. 2015 Feb 6;16:1. doi: 10.1186/s12860-015-0047-7 (PMC4340280; doi:10.1186/s12860-015-0047-7)

Additional file 4. BACE457 and BACE457$_{DHF}$ luminal parts predicted ribbon models. Models were prepared by Geno3D server [36], proteins were visualized in the PyMOL [64].

BACE457

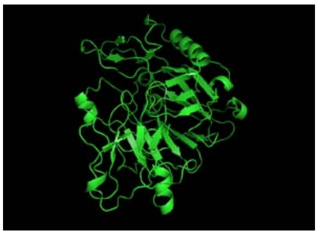

BACE457 DHF

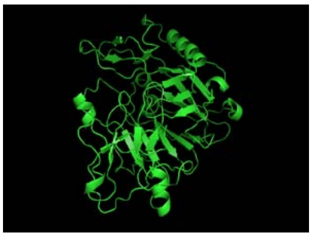

Supplement: Additional file 4: — BACE457 and BACE457 DHF luminal parts predicted ribbon models. [file 12860_2015_47_MOESM4_ESM.pdf]
